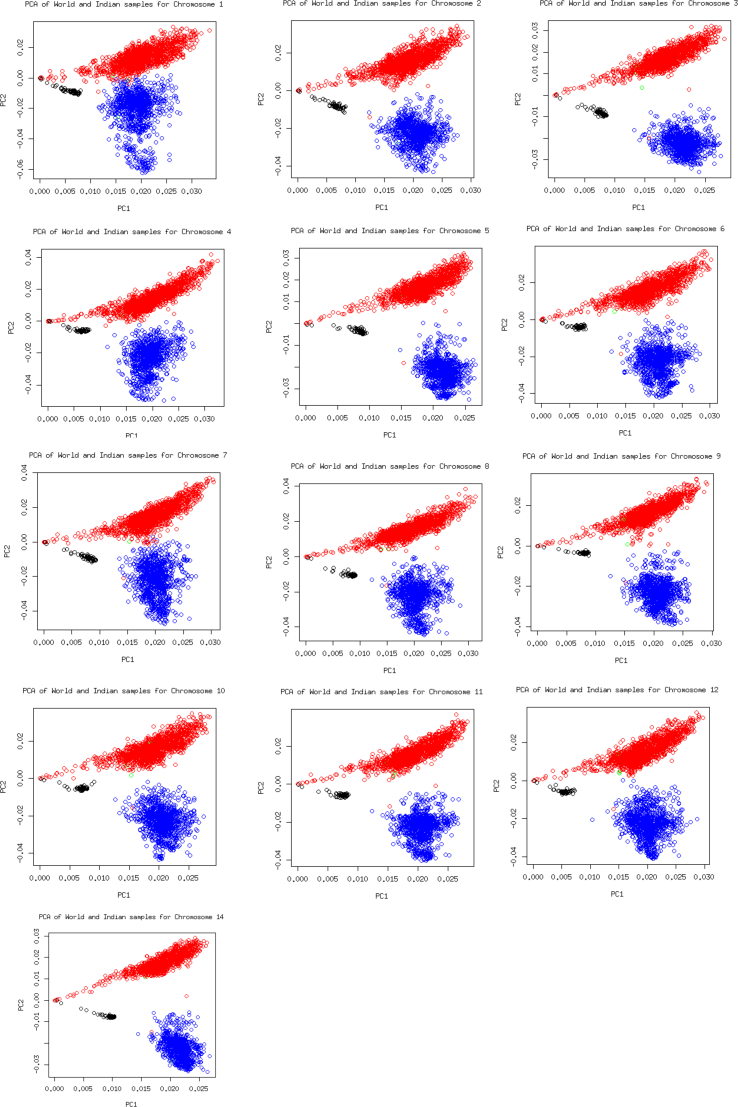# Genomic analysis of Indian isolates of *Plasmodium falciparum*: Implications for drug resistance and virulence factors

**DOI:** 10.1016/j.ijpddr.2023.05.003

**Published:** 2023-05-29

**Authors:** Deepak Choubey, Bhagyashree Deshmukh, Anjani Gopal Rao, Abhishek Kanyal, Amiya Kumar Hati, Somenath Roy, Krishanpal Karmodiya

**Affiliations:** a Department of Technology, Savitribai Phule Pune University, Pune, India; b Department of Biology, Indian Institute of Science Education and Research, Dr. Homi Bhabha Road, Pashan, Pune, 411008, Maharashtra, India; c Department of Medical Entomology, Calcutta School of Tropical Medicine, Kolkata, West Bengal, India; d Department of Human Physiology, Vidyasagar University, Paschim Medinipur, West Bengal, India

**Keywords:** Malaria, *Plasmodium falciparum*, Indian isolates, Artemisinin resistance, PfKelch13 mutations, Genomics

## Abstract

The emergence of drug resistance to frontline treatments such as Artemisinin-based combination therapy (ACT) is a major obstacle to the control and eradication of malaria. This problem is compounded by the inherent genetic variability of the parasites, as many established markers of resistance do not accurately predict the drug-resistant status. There have been reports of declining effectiveness of ACT in the West Bengal and Northeast regions of India, which have traditionally been areas of drug resistance emergence in the country. Monitoring the genetic makeup of a population can help to identify the potential for drug resistance markers associated with it and evaluate the effectiveness of interventions aimed at reducing the spread of malaria. In this study, we performed whole genome sequencing of 53 isolates of *Plasmodium falciparum* from West Bengal and compared their genetic makeup to isolates from Southeast Asia (SEA) and Africa. We found that the Indian isolates had a distinct genetic makeup compared to those from SEA and Africa, and were more similar to African isolates, with a high prevalence of mutations associated with antigenic variation genes. The Indian isolates also showed a high prevalence of markers of chloroquine resistance (mutations in Pfcrt) and multidrug resistance (mutations in Pfmdr1), but no known mutations associated with artemisinin resistance in the PfKelch13 gene. Interestingly, we observed a novel L152V mutation in PfKelch13 gene and other novel mutations in genes involved in ubiquitination and vesicular transport that have been reported to support artemisinin resistance in the early stages of ACT resistance in the absence of PfKelch13 polymorphisms. Thus, our study highlights the importance of region-specific genomic surveillance for artemisinin resistance and the need for continued monitoring of resistance to artemisinin and its partner drugs.

## Graphical abstract

**Figure:**
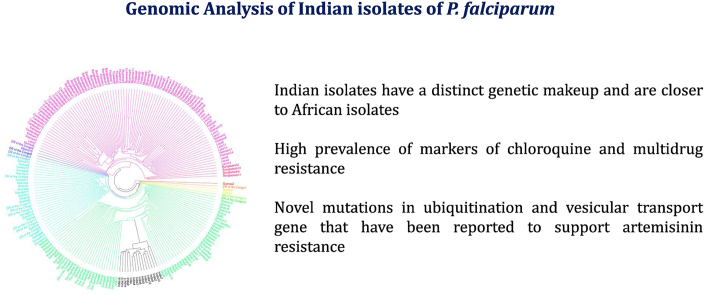

## 1 Introduction

Malaria continues to pose a significant public health challenge in many developing countries, with *Plasmodium falciparum* being the primary cause of malaria-related deaths. In 2021, approximately 247 million malaria cases and 625,000 malaria deaths were reported worldwide, with sub-Saharan African countries being the most affected followed by Southeast Asian (SEA) countries (OrganizationWorld Health, 2022). Resistance to conventional antimalarial drugs such as chloroquine, sulfadoxine-pyrimethamine, mefloquine, and atovaquone is a major challenge in malaria control (Clyde and Shute, 1954; Babiker et al., 2001; Cowman et al., 1994; Durrand et al., 2004; Maberti, 1960; Phillips et al., 1996; Peterson et al., 1988). Currently, artemisinin derivatives and partner drugs in the form of artemisinin-based combination therapy are considered as the first line of defense against malaria (White and Olliaro, 1996; Dhorda et al., 2021). However, 2009 onwards, alarming incidents of resistance against artemisinin have been reported in Southeast Asia (Miotto et al., 2013; Noedl et al., 2010; Takala-Harrison et al., 2015), which is the epicenter for emergence of anti-malarial drug-resistance. The trajectory of artemisinin resistance spread followed a historical trend of spread of antimalarial resistance and reports of artemisinin resistance emerged from India (Das et al., 2018, 2021; Chakrabarti et al., 2019; Mishra et al., 2016) and Africa (Kayiba et al., 2021; Balikagala et al., 2021).

Artemisinin-resistant parasites are characterized by slow growth and reduced drug-susceptibility at the ring stage of asexual growth (Tilley et al., 2016). Population genomics studies have identified mutations in PfKelch13 gene as a molecular marker for artemisinin resistance (Ariey et al., 2014; Miotto et al., 2015; Wicht et al., 2020). Moreover, several studies also indicated transcriptional rewiring particularly oxidative stress and protein damage responses as major contributor to the artemisinin resistance (Mok et al., 2015; Rocamora et al., 2018; Rawat et al., 2021). Recently, many reports from Southeast Asia and Africa indicated PfKelch13 independent emergence of artemisinin resistance suggesting alternative pathways for acquiring resistance (Das et al., 2021; Nima et al., 2022; Takala-Harrison et al., 2015; Mukherjee et al., 2017; Ndwiga et al., 2021). These multiple independent mechanisms of artemisinin resistance suggest that there may not be a single “universal identifier” of artemisinin resistance but a number of them each specifically built/selected upon a complicated genetic background shaped by years of differential evolution in the field. Furthermore, considering the sheer abundance of cellular targets for artemisinin in the cell, and the fitness cost associated with the PfKelch13 mutations, numerous “background mutant genes” may be required before resistance is manifest (Hott et al., 2015; Nair et al., 2018). Recent reports also suggest the possibility of co-existing mutations in the background genome that assist resistant parasites in survival (Miotto et al., 2015; Rawat et al., 2022; Ray et al., 2022) or can be independently responsible for artemisinin resistance (Chakrabarti et al., 2019; Das et al., 2021; Mishra et al., 2016; Nima et al., 2022). Taken together, it suggests that though PfKelch13 is strongly associated with the artemisinin tolerance, there are possibly other genetic determinants associated with the decreased artemisinin tolerance in isolates from Africa and India.

In this study, we report the whole genome sequencing of 53 *P. falciparum* parasite isolates from the Kolkata region of India, a site from where the first definitive incident of artemisinin resistance was reported (Das et al., 2018). It is previously estimated that 10–15% of falciparum malaria cases show artemisinin resistance in Kolkata region while little is known about its emergence and molecular markers (Das et al., 2018, 2021; Nima et al., 2022). Through the whole genome sequencing we explored the landscape of parasite genome associated with drug resistance in India with key focus on non-synonymous mutations present in genes involved in resistance. Our findings reveal the presence of a novel L152V mutation in the PfKelch13 gene in one of the samples and the absence of distinctive SNPs in these isolates. Additionally, we have identified a novel set of mutations in genes involved in pathways associated with artemisinin processing, including vesicular transport and ubiquitination. Our study provides important insights into the genetic makeup of Indian isolates of *P. falciparum* and highlights the need of system-wide analysis, characterization and validation of resistance marker genes to combat malaria.

## 2 Results

### 2.1 Indian isolates of *P. falciparum* show a distinct genetic makeup compared to other Asian and African isolates

There is a need for a thorough understanding of the genome of the malaria parasite from various endemic regions, particularly in areas where drug resistance has emerged. Samples from these regions can be analyzed to understand the dynamics of host-pathogen interactions, specific characteristics in their genomic architecture, and potential markers of drug resistance. It is known that the endemic history of drug use in a population can lead to the evolution of drug tolerance. For example, artemisinin resistance in many regions has been acquired through human migration and through the continuous use of artemisinin derivatives (Müller and Hyde, 2010). It is important to identify the closest lineages of the parasites and understand their similarities and differences with endemic regions in nearby countries.

**Fig. 1:**
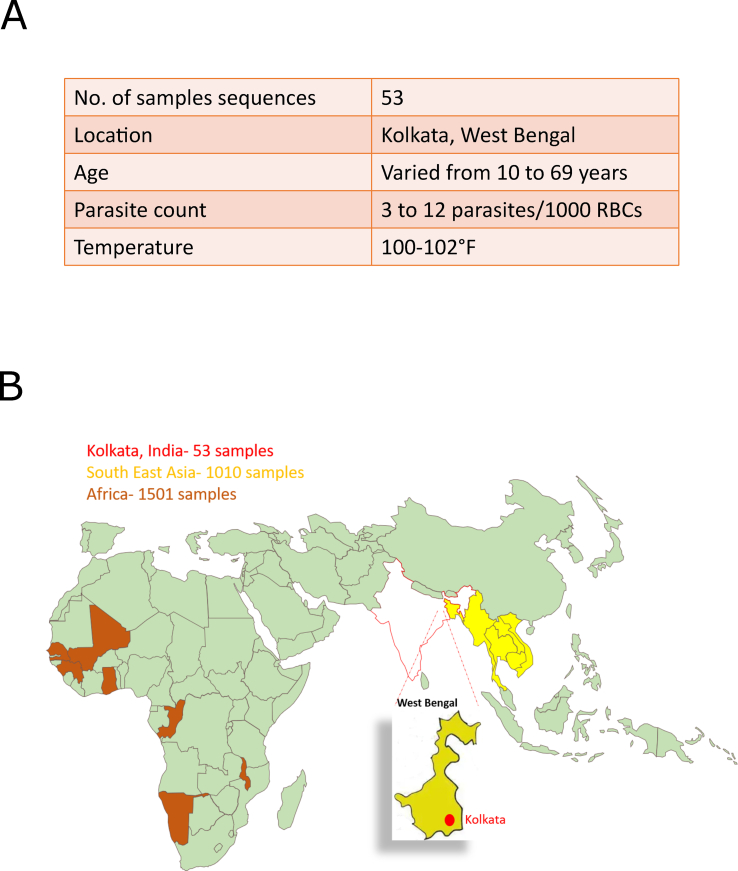
**Sample information and site for sample collection in India. (A)** Table representing patient demographics for 53 samples collected from Kolkata, India. **(B)** Map representing location of sample collection site of Kolkata (bordering Bangladesh) represented in red. Genome sequencing data downloaded from MalariaGen for 1010 samples from SEA (countries colored in yellow) and 1501 samples from Africa (countries colored in brown). (For interpretation of the references to color in this figure legend, the reader is referred to the Web version of this article.)

**Fig. 2:**
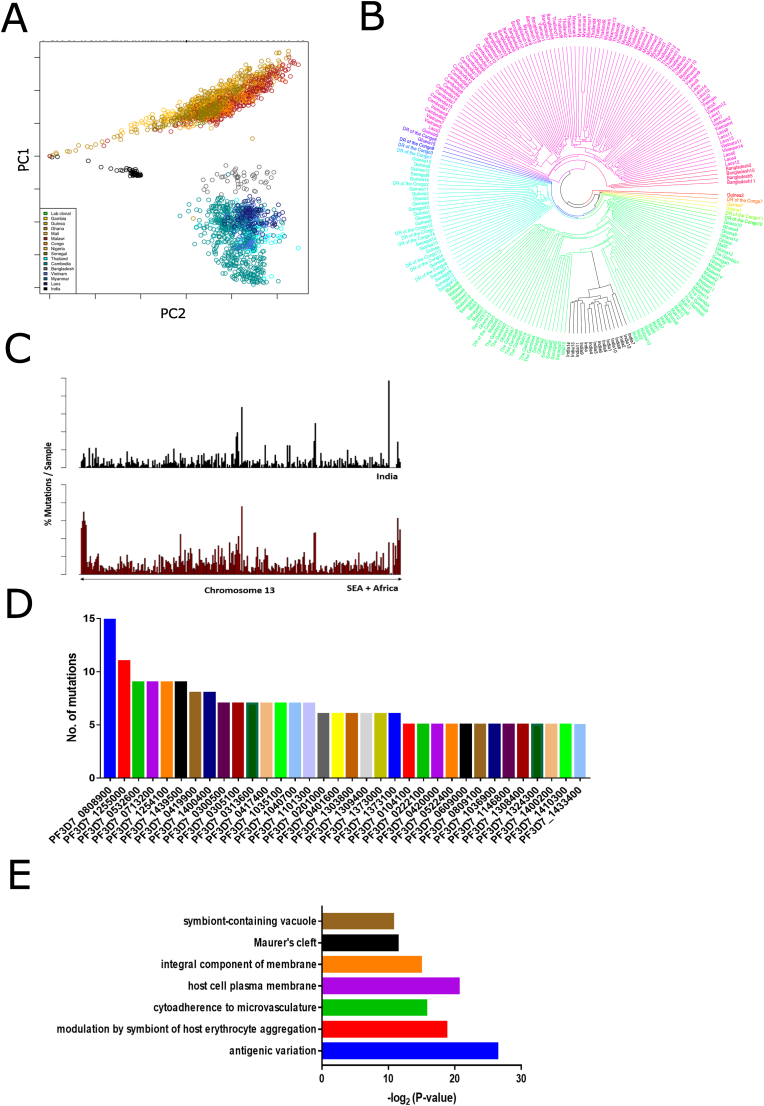
**Comparison of the *P. falciparum* genomes from India, Southeast Asia, and Africa. (A)** A principal component analysis (PCA) plot of chromosome 13 showing distinct clustering of Indian isolates (represented in black) in comparison with isolates from other countries from Southeast Asia and Africa. **(B)** Phylogenetic analysis using 15 representative genomes per country depicting the closeness of the Indian isolates to African isolates. **(C)** A graph showing the percentage of single nucleotide polymorphisms (SNPs) across genes of chromosome 13 in Indian isolates (upper panel) compared to samples from Southeast Asia and Africa (lower panel). **(D)** A graph showing the number of mutations in 34 genes identified as having higher mutations (more than 5 per gene). **(E)** Gene ontology analysis of the 34 identified genes, indicating enrichment of antigenic variation genes.

We performed whole genome sequencing of 53 *Plasmodium falciparum* isolates from Kolkata, West Bengal, India and compared them to isolates from Southeast Asia (SEA) and Africa (Fig. 1A and B). A principal component analysis (PCA) was performed to investigate the relatedness between the Kolkata samples and the global endemic region dataset, which includes a total of 2570 genomes from 15 countries (Fig. 2A and Supplementary Fig. S1). The PCA analysis of all 14 chromosomes showed distinct clusters of parasite genomes from different geographic regions, as expected, with the cluster representing Indian isolates showing a unique position compared to global isolates (Supplementary Fig. S1). The Indian isolates were farther from those in SEA but closer to African isolates (Fig. 2A). The phylogenic analysis of 15 representative samples from each country also corroborates with the unique positioning of India as compared with other isolates (Fig. 2B). Similar clustering has been observed for *Plasmodium vivax*, with isolates from India and Sri Lanka showing relatedness to South African isolates (Daron et al., 2021), possibly due to human migration between the Indian subcontinent and Africa. Although the area of sample collection (Kolkata, West Bengal) lies on close boundaries with SEA countries where human migration is high, contradicting to it we see significant relatedness with African isolates in our study. Thus, there is a distinct genetic identity of Indian isolates compared to African and other Asian isolates.

### 2.2 Indian isolates have abundance of specific mutations associated with antigenic variation genes

To further investigate the genetic differences between Indian isolates and those from other regions, we compared the mutation burden in parasites from India and other geographical locations. For this analysis, we divided the chromosomes into 10k base-pair bins and counted the number of mutations present (Fig. 2C). Isolates from Southeast Asia and Africa had relatively high polymorphism in all chromosomes compared to Indian isolates, particularly in chromosome 13, which is known to play a vital role in artemisinin resistance. To identify genes with high polymorphism in Indian isolates specifically, we conducted an analysis of single nucleotide polymorphisms (SNPs) to identify genes exhibiting high polymorphism. We partitioned the SNP dataset to identify mutations present in more than 75% of Indian isolates and less than 25% of other global isolates (adapted from a previous study by (Rawat et al., 2022)). From this analysis, we identified 1581 mutations that were specific to Indian isolates, spread across 922 genes (Supplementary Table S2). We examined genes with a minimum of 5 different mutations per gene and found 34 such genes (Fig. 2D). Interestingly, one of the gene families with the highest polymorphism in Indian isolates is the virulence gene family (Fig. 2E). Virulence genes play a significant role in falciparum malaria infection, contributing to the evasion of the immune system and splenic clearance through antigenic variation, which can modulate the severity of *P. falciparum*-caused malaria. These antigenic variation genes have specificity for host endothelial cell receptors and allow the parasites to remain adhered in the microvasculature (Smith et al., 2001). Since virulence genes are known to be polymorphic and prone to recombination events, it is possible that Indian isolates have a unique antigenic gene repertoire. This could have a significant impact on understanding the virulence and severity of *P. falciparum* malaria in India and have implications for the development of therapies targeting virulence genes.

### 2.3 Indian isolates are defined by presence of multidrug resistance markers but devoid of markers for artemisinin resistance

**Fig. 3:**
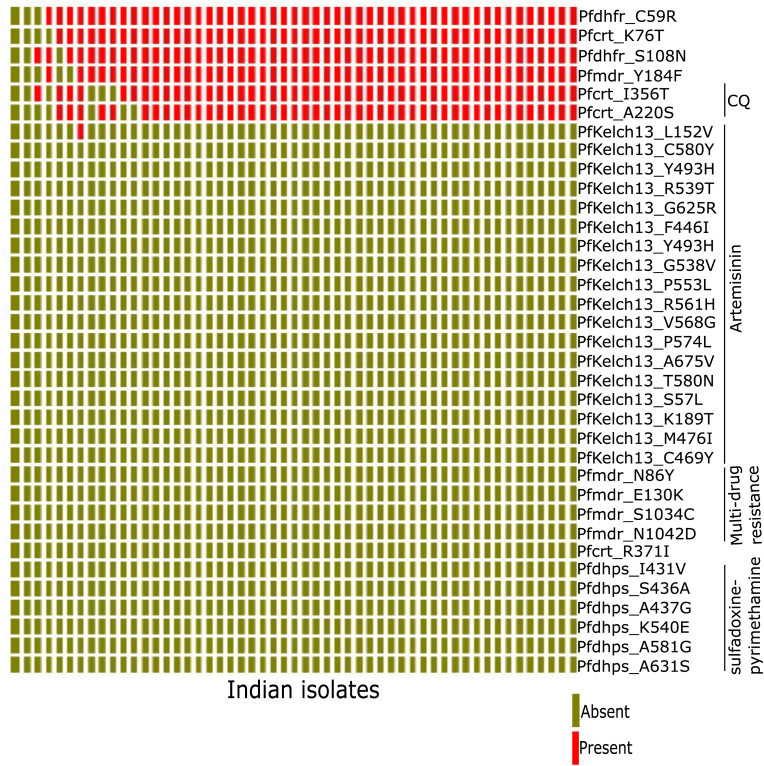
**Status of drug-resistance marker genes in Indian isolates of *P. falciparum*.** Plot depicting presence or absence of mutations in genes known for drug-resistant marker in Indian isolates. The known markers of artemisinin resistance, mutation in PfKelch13 gene were not found in Indian isolates.

We further screened the genetic landscape of the 53 Indian isolates for markers of known drug resistance. We were principally interested in the status of drug-resistance by monitoring the mutations in the candidate genes; *Plasmodium falciparum* chloroquine resistance transporter (pfcrt), *Plasmodium falciparum* multidrug resistance protein 1 (pfmdr-1) for chloroquine; pfmdr-1 for amodiaquine, and dihydropteroate synthase (pfdhps) and dihydrofolate reductase (pfdhfr) for pyrimethamine and sulfadoxine resistance, respectively and PfKelch13 for artemisinin resistance. Our analysis showed mutation of L152V in PfKelch13 gene while no mutations were detected in definitive SNPs (C580Y, R539T and Y493H) associated with artemisinin resistance across the 53 genomes analyzed (Fig. 3). Among the five definitive markers of chloroquine resistance (K76T, A220S, Q721E, I356T, R371I), we detected three (K76T, A220S, I356T) in Pfcrt gene (Fig. 3). This reinforces the prevalence of chloroquine resistant *P. falciparum* in Indian isolates, although the phenotypic data for these lines is not available to us. We also looked for the following mutations in Pfdhfr: C59R, N51I, S108N, I164L and found C59R and S108N present in Indian isolates. For Pfdhps we looked for I431V, S436A, A437G, K540E, A581G, and A631S none of which are present in Indian isolates. Additionally, only one marker of multidrug resistance was identified in Indian isolates (pfmdr-1: Y184F) (Fig. 3). Taken together, our results align with the drug resistance observed in the field against classical drugs used in anti-malarial therapy.

**Fig. 4:**
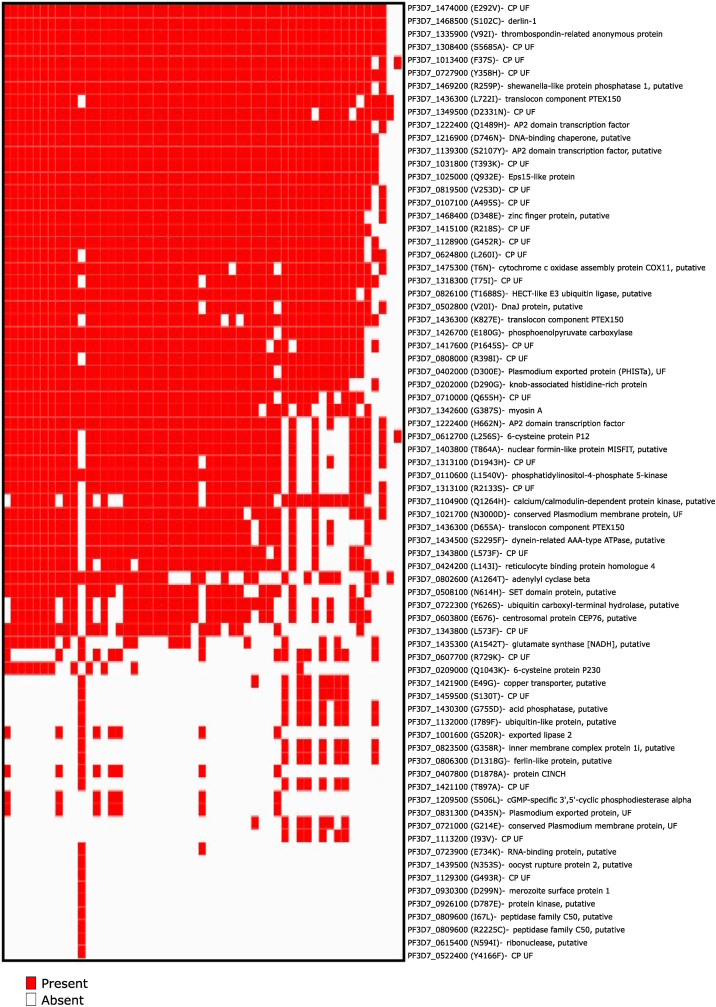
**Analysis of other mutations shown to be associated with the artemisinin resistance.** A binary heatmap showing the presence of mutations in Indian isolates that has overlap with PfKelch13 mutated artemisinin-resistant samples previously analyzed in Rawat et al. (2022). Mutations in Pfderlin-1 and cGMP-specific 3′5′- cyclic phosphodiesterase alpha are present in Indian samples, which are previously seen to be present in endemic regions of Cambodia during year 2008–2011 having early signs of ACT resistance. CP and UF stand for Conserved Plasmodium protein and Unknown function respectively.

Earlier reports have identified 10–15% resistance (underscored by delay in parasite half-life clearance of >5 h and persistence of parasitemia at 72 h after ACT using ex-vivo RSA 0–3h) in *P. falciparum* caused malaria patients from Kolkata and nearby regions (Das et al., 2018, 2021; Nima et al., 2022). Importantly, in these cases the emergence of artemisinin resistance is independent of PfKelch13 mutations (Ghanchi et al., 2021; Nima et al., 2022). This indicates a possibility of PfKelch13 independent artemisinin resistance by a novel set of mutations. Since there are ample reports suggesting ACT resistance independent of PfKelch13 (Das et al., 2021; Ghanchi et al., 2021; Nima et al., 2022), we looked into a list of mutations of genes that have a high association probability in ACT resistant samples (Rawat et al., 2022). We then compared the list of genes having mutations in Indian isolates with that of genes defined to be co-existing in PfKelch13 mutated/artemisinin resistance isolates (Rawat et al., 2022). We saw an overlap of 74 mutations present in Indian isolates with a few genes (conserved proteins of unknown function) prevalent in 51 samples overall (Fig. 4). Importantly, we see mutation in Pfderlin-1 gene (PF3D7_1468500) known to be involved in polyubiquitination and cGMP-specific 3′5′- cyclic phosphodiesterase alpha (PF3D7_1209500). These have been prevalent in endemic regions of Cambodia during year 2008–2011 having early signs of ACT resistance (Dwivedi et al., 2017). Since pathways such as vesicular transport, ubiquitination, protein degradation, ferredoxin pathways are known to be involved in mechanism of artemisinin action/resistance we focus on genes involved in these pathways. We observed that HECT-like E3 ubiquitin ligase (PF3D7_0826100), putative ubiquitin carboxyl-terminal hydrolase (PF3D7_0722300), putative ferlin-like protein (PF3D7_0806300, involved in vesicular fusion) had mutations (Obrova et al., 2019; Simwela et al., 2020). These mutations might alternatively promote the evolution of ACT resistance in the Indian subpopulation in the future. In summary, our data validates the existence of classical mutations known to be associated with resistance to a wide variety of antimalarial compounds but does not show presence of PfKelch13 mutations associated with artemisinin resistance. Instead, we observe novel/alternative mutations in genes associated with ubiquitination and vesicular transport processes. There is no concrete evidence of correlation of these mutations with artemisinin resistance in the field and with a lack of phenotypic data (ring stage survival assay to indicate the status of artemisinin resistance in our isolates), it is still an open challenge to identify the definitive drivers of PfKelch13-independent artemisinin resistance in the region.

## 3 Discussion

Our genome-wide comparison of global isolates with Indian strains of *P. falciparum* reveals the unique genetic makeup of the latter. This uniqueness is also observed at the individual chromosome level, suggesting a strong regional identity of Indian isolates compared to those from other regions around the world. The distinct genetic makeup of Indian isolates may be due in part to the endemic nature of malaria in certain hot-spots in the Indian subcontinent, which has likely preserved the genetic diversity of the parasites in these areas. Additionally, cases of malaria in urban centers in India are often promptly detected and treated, while cases in rural areas may go undiscovered, potentially limiting the spread of *P. falciparum* and preserving its genetic diversity. This falls in line with observations from across the globe that regional isolated populations are largely distinct from each other (Kumar et al., 2016).

Our analysis of mutations specific to Indian isolates also reveals a high number of mutations in genes associated with host-parasite interactions (members of the multi-gene family), which are known to be highly region-specific and influenced by host pressures. This suggests that unique host-specific factors may have driven the evolution of distinct mutations in these genes. These findings are important in the context of the link between virulence genes and host immunity, including the development of host immunity and the effectiveness of vaccination. Thus, the unique build-up of mutations in virulence genes in Indian isolates may have implications for vaccine development policies specific to these regional isolates.

Multidrug resistance, particularly against chloroquine, sulfadoxine-pyrimethamine, and mefloquine, is prevalent in different regions of India, and drug policies are periodically reviewed in response. Our data supports this by demonstrating the prevalence of Pfcrt and Pfmdr1 mutations in Indian isolates. However, we did not detect PfKelch13 mutations, which are experimentally validated determinants of artemisinin resistance. Similar findings have been reported for samples from districts in Bangladesh where ACT resistance is observed, but PfKelch13 polymorphisms are absent (Nima et al., 2022). Reports of artemisinin resistance in India are sporadic and mostly from the north-eastern regions of the country. Our data suggest that although mutations in PfKelch13 are absent, mutations in genes involved in processes such as ubiquitination and vesicular transport may act as novel alternative mutations supporting future evolution of artemisinin resistance. This indicates the possibility of unique mutations determinative of artemisinin resistance, in addition to PfKelch13. However, further analysis in the form of genome-wide association studies comparing phenotypic drug-sensitivity data with genotype information will be necessary to make such inferences. Overall, our study highlights the distinct features of Indian *P. falciparum* isolates, including the genetic basis for widespread chloroquine resistance (along with the absence of artemisinin resistance). It also emphasizes the potentially distinct emergence of mutations in multigene families specific to host-parasite interactions in these isolates, which may be relevant to vaccination studies.

## 4 Material and methods

### 4.1 Study site

Patient blood samples N = 81 was collected during October to December 2019 after detecting *P. falciparum* ring stage with parasite count of 3–12/1000 RBCs in smear preparations. At the time of collection patients reported presence of cyclic fever of 100–102 °C with chills (alternative days) and headache for 2–7 days. Sample collection was done at Gautom Laboratory and Imaging Center, Central Kolkata (endemic region wherein first report of artemisinin resistance in India was reported) for diagnosis and remaining blood was used for sequencing purpose. All patients diagnosed with malaria were provided with proper treatment and cured.

### 4.2 Patient sample preparation (leukocyte filtration)

The *Plasmodium falciparum* whole blood samples were leuco-depleted using a cellulose column filtration. First, the serum was separated by centrifugation. The pellet was washed thrice with 1X PBS and resuspended in 1 mL of 1X PBS. For preparing the cellulose column, 3 vol of cellulose powder per 1 volume of whole blood was packed into a column and equilibrated with 2 column volumes of 1X PBS. The resuspended sample was then loaded onto the column and the flowthrough (infected RBCs) was collected. The infected RBCs were washed thrice with 1X PBS and stored until isolation of genomic DNA.

### 4.3 Genomic DNA isolation and selective whole genome amplification (sWGA)

Genomic DNA was isolated from the *Plasmodium falciparum* blood samples using the QIAmp DNA Blood Mini Kit following the manufacturer's instructions. The isolated genomic DNA was subjected to sWGA following a previously published protocol (Oyola et al., 2016). All the reactions were carried out in a UV Cabinet for PCR operations to avoid any contamination and the amplification was performed in a 0.2 mL 96-well PCR plate (Bio-Rad). Briefly, a minimum of 10 ng of genomic DNA was added to a 50 μL reaction containing 1X EquiPhi29 DNA polymerase reaction buffer (Thermo Scientific), 1X nuclease-free BSA (Genei), 2.5 μM of each primer, 1 mM dNTP (New England Biolabs), 10 units of EquiPhi29 DNA Polymerase (Thermo Scientific) and nuclease free water. The plate was sealed and placed in a Thermal Cycler (Bio-Rad) with the cycling conditions of a step-down isothermal PCR: 35 °C for 5 min, 34 °C for 10 min, 33 °C for 15 min, 32 °C for 20 min, 31 °C for 30 min, 30 °C for 16 h followed by heat-inactivation of the Phi29 at 65 °C for 15 min and cooling to 10 °C. The sWGA products were then cleaned up using Agencourt Ampure XP beads (Beckman Coulter) following the manufacturer's instructions: 1.8 vol of beads per 1 volume of sample were mixed and incubated for 5 min at room temperature. After incubation, the tube containing bead/DNA mixture was placed on a magnetic rack to capture the DNA-bound beads while the unbound solution was discarded. Beads were washed twice with 200 μl of 70% ethanol and the bound DNA was eluted with 40 μL of nuclease free water. Cleaned amplified DNA products were used to prepare a library for whole genome sequencing. Primers used for the sWGA reactions were the same as (Oyola et al., 2016).

### 4.4 Protocol for Illumina sequencing

Out of 81 samples, 4 were omitted as they had high human DNA contamination. Whole genome sequencing was carried out for 77 sWGA samples of *Plasmodium falciparum* genomic DNA. Standard libraries of 200–500 bp DNA fragments were prepared using the Nextera XT DNA Library Preparation Kit (96 samples) following the manufacturer's instructions. In brief, 10–100 ng of DNA samples were fragmented, barcoded using the Nextera XT Index Kit (24 indexes, 96 samples) and purified. This was followed by fragment size analysis using the Agilent 2100 Bioanalyzer system and concentration analysis using a Qubit 4.0 fluorometer. Libraries were then pooled and sequenced on the Illumina NextSeq550 System using the mid-output 150 bp paired/single-end sequencing chemistry. We sequenced 77 samples and 53 of them had coverage above 20X which were taken ahead for analysis.

### 4.5 Data analysis

For this study, we analyzed whole genome sequencing data procured from the Pf3K (Project, 2016), Malaria Gen which represent 2517 samples primarily from 15 countries in Asia and Africa (Project, 2016). We additionally merged sequencing data from 53 in-house sequenced Indian isolates for a total of 2570 data points. We first did the mapping of whole genome sequences using bwa with the default parameters. SNP calling for the Indian isolates was performed with GATK v4.0 pipeline followed by annotation for both Indian and Pf3K samples with snpEff. We marked the duplicate reads MarkDuplicate tool from Picard with the default options. HaplotypeCaller was used to for calling the variants with default parameters. We than extracted the SNPs with non-synonymous mutation effect and generated the matrix out of it for the further analysis. Additional analysis (matrix development, PCA, etc.) was done using custom Perl and R scripts. We created a combined matrix for SNP coordinates across all samples, where 0 and 1 denote the absence and presence respectively of an SNP at a particular coordinate for a specific sample. This exercise was additionally repeated for all chromosomes individually to develop chromosome specific PCA profiles for all the samples.

### 4.6 Data access

The genome sequencing data is deposited to Sequence Read Archive (SRA) under BioProject ID PRJNA911475.

## Ethics statement

We are indeed thankful to the Gautam Laboratories and Imaging, Kolkata, India (NABL accredited laboratory, ISO 15189:2007-M-0423) for their Continuous help. Under their supervision, in vivo tests were done. We are undoubtedly grateful to all the patients who participated in the work. The approval to perform whole genome sequencing of malaria samples has been granted by the Institutional Biosafety Committee (IBSC) of Indian Institute of Science Education and Research Pune.

## Authors' contributions

DC, BD, AGR and AK designed, performed experiments, and analyzed data. AKH and SR collected samples and performed initial screening of samples. AK and AGR processed the samples and generated NGS data. BD, DC, SR and KK wrote the manuscript. KK planned, coordinated, and supervised the project. All authors read and approved the final manuscript.

## Funding

This work was partially supported by the DBT- Infectious Disease Biology division (BT/PR41408/MED/29/1547/2020) from the Government of India to KK. The funders had no role in study design, data collection and analysis, decision to publish, or preparation of the manuscript.

## Declaration of competing interest

The authors declare that they have no conflict of interest.

## Figures and Tables

**figs1:**